# An accurate method of measuring shoulder sling compliance: a validation study

**DOI:** 10.1186/s12891-021-04396-1

**Published:** 2021-06-07

**Authors:** Anshum Sood, Ashley Klein, Samir Kaveeshwar, Derek L. Jones, Grant Duvall, James Paul Hovis, Tristan B. Weir, Blessing Enobun, S. Ashfaq Hasan, R. Frank Henn, Jonathan D. Packer, Mohit N. Gilotra

**Affiliations:** 1 Department of Orthopaedics, University of Maryland School of Medicine, 100 Penn Street, Room 540D, Baltimore, Maryland 21201 USA; 2 Department of Orthopedics, University of Arizona College of Medicine, Phoenix, Arizona USA

**Keywords:** sling, sling adherence, rotator cuff, brace, rehabilitation

## Abstract

**Background:**

The effect of postoperative shoulder sling compliance on surgical outcomes is unknown. The goal was to determine an accurate method to measure sling compliance. We compared volunteer recorded sling wear time with temperature-based sensors to monitor sling compliance.

**Methods:**

Data loggers sutured at three locations measured heat generated in 15-minute intervals. Slings wearers logged sling wear to accurately cross-reference with temperature sensors. Secondary experiments analyzed whether surrounding ambient temperature can be discerned from actual sling wear. We created an algorithm to describe actual sling wear time as a function of heat recorded and calculated percent wear accuracy.

**Results:**

The modified sling was worn for 172 h. The algorithm modeled sling on/off times by analyzing cutoff temperatures. Diagnostic accuracy was >99 % for the three locations, with no statistically significant differences among them. Compared with sling wear, ambient temperature took longer to reach critical temperature values determined by the algorithm, helping distinguish compliance from false positives.

**Conclusions:**

The described algorithm can effectively quantify shoulder sling wear time based on heat-generated sensor readings. False positives from ambient temperature are minimal. This measurement method could be used to study the relationship between postoperative sling use and functional outcomes after shoulder surgery.

**Supplementary Information:**

The online version contains supplementary material available at 10.1186/s12891-021-04396-1.

## Background

Adherence to medical treatment can be defined as the extent to which patients follow treatment protocols prescribed by their physicians. Adherence rates are higher for acute conditions than for chronic illnesses. Although adherence rates are difficult to measure, higher adherence rates usually correlate to better clinical outcomes [1]. This trend is commonly noted in medication regimens for diseases such as cancer, hypertension, schizophrenia, and many others [2–4].

In orthopaedics, immobilization is prescribed following closed reduction of fractures and either postoperatively or as the primary intervention to manage idiopathic conditions such as scoliosis and clubfoot [5, 6]. After rotator cuff repair, a sling is used to prevent strain at the tendon repair site. Excessive strain can lead to failure of the repair [7–9]. It is unknown to what extent patient adherence to the rehabilitation protocol affects functional outcome. One study relied on patient surveys to evaluate adherence to the postoperative protocol; however, that method of collecting data is likely inaccurate [10].

Previous studies that have used temperature sensor technology to objectively measure brace adherence found that self-reported adherence is significantly higher than the temperature-recorded adherence rates [11, 12]. In those studies, the temperature sensors used in braces were in close contact with patients’ bodies. However, shoulder slings have a looser fit, and it has not been reported whether temperature sensors can accurately measure sling use. Therefore, the purpose of our study was to investigate the accuracy of temperature sensors placed in shoulder slings. We additionally evaluated whether the temperature sensor could discern the difference between a hot environment and body temperature. We hypothesized that the temperature sensors would accurately measure sling wear and would accurately discern the difference between a hot environment and body temperature.

## Methods

### Validation overview

The Onset series of data loggers (HOBO MX2201; Onset Computer Corporation, Bourne, MA) were fitted onto three locations on each of four DonJoy UltraSling (DonJoy Performance, Dallas, TX) shoulder slings, which are routinely provided to patients postoperatively. A trial was then conducted with the retrofitted slings in which they were worn throughout the day and at night by volunteers to simulate the patient experience, including removing and re-applying the sling to best emulate exercise sessions and other instances in which a patient might remove the sling (e.g., for personal hygiene). The subjects were asked to wear the sling as much as possible but were free to remove the sling to perform daily activities. This provided multiple sling wear sessions throughout the day to illustrate temperature differences when the sling was on or off the volunteer. We used healthy volunteers in this validation study because of concern that patients on pain medicine would not be reliable participants. Volunteers ranged between the ages of 25 to 32 years (three males, one female), had no significant past medical history, and had a body mass index in the normal range. All participants had a body temperature in the normal range and were not febrile during the study period. The four participants kept detailed timetables of actual sling wear time to compare with the wear time detected by the temperature sensors. The volunteers understood the study aims and were not given a specific sling wear schedule to avoid bias when they logged their time in the sling. We considered the logged sling time the “actual wear time.” When the sling was not in use, we asked volunteers to leave the sling in an environment at room temperature. The sling was worn in both winter and summer months.

### Data loggers

**Fig. 1:**
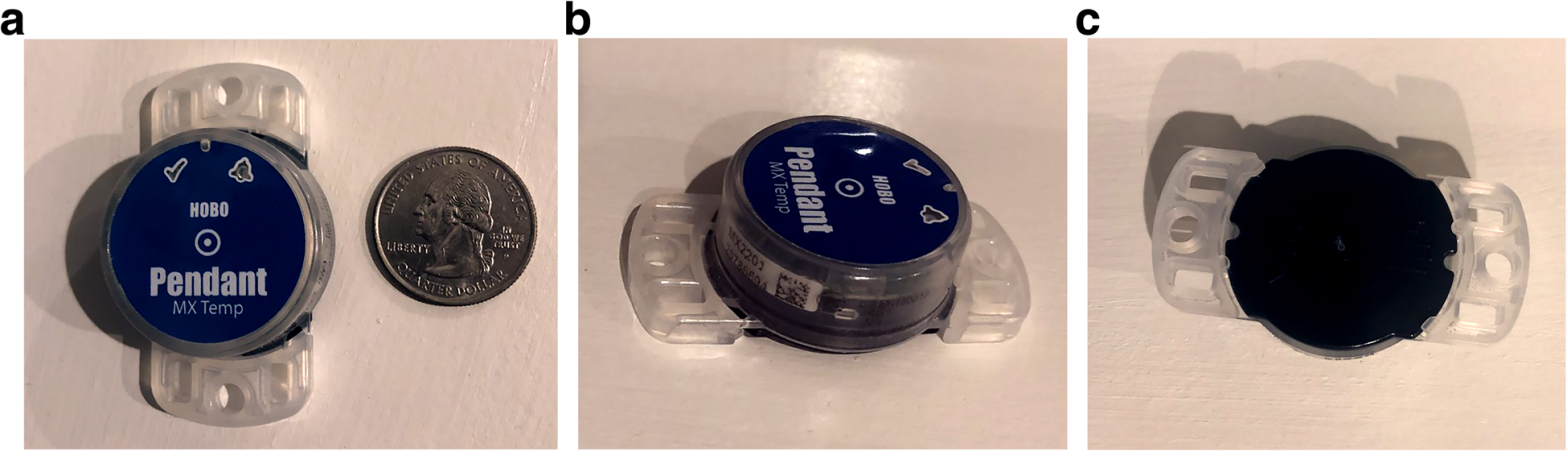
Front (**a**), side (**b**), and back (**c**) views of the Onset Hobo MX2201 (Onset Computer Corporation, Bourne, MA)

The Onset HOBO MX2201 (Onset Computer Corporation, Bourne, MA) data loggers were used to track shoulder sling wear. The data loggers are compact (dimensions, 3.35 × 5.64 × 1.8 cm; weight, 12.75 g), battery-powered devices (battery life 1–2 years) that feature an internal microprocessor, data storage, and sensors that can measure contact temperature readings with up to 0.5° Celsius (C) of accuracy (Fig. 1). The device can be configured to record temperature readings at different time intervals. The data logger was set to record temperature readings every 15 min to ensure that the memory capacity of the device was not exceeded during a period of >3 months. Data is transferred from the sensors wirelessly via Bluetooth 4.0 technology.

### Sensor locations

**Fig. 2:**
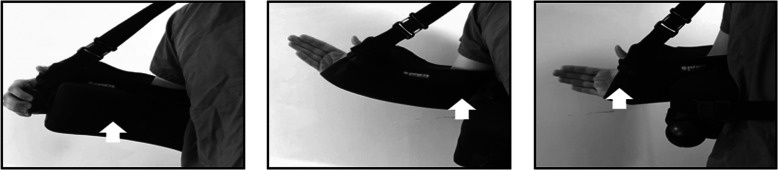
Arrows indicate the three sensor locations: (**a**) inner aspect of bolster at abdomen; (**b**) inner aspect of sling at medial elbow; (**c**) inner aspect of sling at palmar surface of carpometacarpal joint

Data loggers were manually sutured into three locations on each shoulder sling: (1) inner aspect of the bolster that is in contact with the abdomen, (2) inner aspect of the sling at the medial elbow, and (3) inner aspect of the sling at the palmar surface of the carpometacarpal joint (Fig. 2). These locations were chosen because they were noted to have more continuous body contact when the shoulder sling was worn. Continuous body contact with the implanted data loggers allows for accurate temperature readings and decreased fluctuations that can occur if the data logger is not flush against the body. Three locations were selected to help inform future studies on the optimal location for sensor placement.

### Ambient temperature control

The ability of the sensor to recognize between body heat while the sling is being worn and ambient heat in an environment was also evaluated. Specifically, we investigated whether the difference between wearing the shoulder sling retrofitted with the temperature sensors could be discerned from storing it in a hot environment. To do this, the retrofitted sling was placed into the trunk of a car during a hot day with the outside temperatures ranging from 80 to 90 °Fahrenheit (F). Afterward, the sensor data were evaluated for differences.

### Algorithm

Based on the temperature sensor data output, an algorithm was created to approximate actual shoulder sling wear time logged by volunteers. The temperatures, timeframes, and conditions in the algorithm were selected to best match the temperature data to the actual sling wear logged by the volunteers. Additional file 1 provides an outline of the proposed algorithm for ease of use.

For a given time point to be considered the start of a wear period, the following conditions must be met. First, either the time point in question must be the first in a consecutive pair of points with a temperature increase of at least 2 °F immediately followed by a temperature ≥83 °F or the time point in question must be the first in a consecutive pair of points with a temperature increase of at least 3 °F immediately followed by another consecutive pair of points with a temperature increase of at least 3 °F followed by a temperature ≥83 °F within 30 min. Second, the temperature must remain ≥83 °F for at least 30 min. Third, the temperature cannot exceed 100 °F.

For a given time point to be considered the end of a wear period, the following conditions must be met. First, the time point in question must be part of an established wear period. Second, the time point in question must be the first of a consecutive pair of time points with a temperature decrease of at least 3 °F. Third, the temperature must decrease below 83 °F within 30 min of the 3 °F drop in temperature.

Additional file 2 provides example data to better illustrate how the algorithm works in practice. The algorithm was then input into a formula on Microsoft Excel to allow for automation of data analysis.

### Statistical analysis

To evaluate for any statistically significant differences in shoulder sling wear time approximation among the three different locations, three unpaired two-sample *t* tests (Microsoft Excel; Microsoft Corporation, Redmond, WA) were run to compare algorithm-generated time approximations among groups. This was done to analyze whether there was a statistically significant difference (*p* < .05) in the time approximations registered by each sensor location on the arm. The sample size of four volunteers is similar to previous studies validating temperature sensors to monitor compliance with bracing for adolescent idiopathic scoliosis, which used three patients [5]. An *a priori* sample size calculation for the correlation between actual and measured sling wear assumed a rho of 0.95, alpha of 0.05, and 80 % power, showing a sample of four patients was adequate.

## Results

The sum of actual sling wear time (i.e., the time logged by the volunteers) was a total of 171.63 h for the four volunteers. The slings were monitored for approximately 1101 h. Per the algorithm described above, the data loggers installed on the bolster, elbow, and wrist areas calculated a total estimated wear time of 167.75, 171.00, and 172.25 h, respectively. The diagnostic accuracies for the bolster, elbow, and wrist areas of the data loggers were 99.5 %, 99.1 %, and 99.3 %, respectively (Table 1).

**Table 1:** Temperature Sensor and Self-Reported Sling Wear Times^a^

	Location^b^		
	**Bolster**	**Medial Elbow**	**Palmar Aspect**
Total actual wear time logged by volunteers, h	171.63	171.63	171.63
Total time sensor on, h	1101.5	1101.5	1101.25
Total estimated time (h) logged by sensor/algorithm	167.75	171.00	172.25
True positives, h	167.25	166.5	168.5
True negatives, h	928.75	924.75	924.75
False positives, h	0.5	4.5	3.75
False negatives, h	5	5.75	4.25
Sensitivity (TP) / (TP + FN), %	97.1	96.7	97.5
Specificity (TN) / (FP + TN), %	99.9	99.5	99.6
Positive predictive value (TP) / (TP + FP), %	99.7	97.4	97.8
Negative predictive value (TN) / (FN + TN), %	99.5	99.4	99.5
Diagnostic accuracy (TP + TN) / (TP + TN + FP + FN), %	99.5	99.1	99.3

^a^Sling wear times are provided as the sum of the four volunteers included in the study.

^b^Bolster, inner aspect of the bolster that is in contact with the abdomen; Medial Elbow, inner aspect of the sling at the medial elbow; Palmar Aspect, inner aspect of the sling at the palmar aspect of the carpometacarpal joint.

True positive, true negative, false positive, false negative, sensitivity, specificity, positive predictive value, and negative predictive value are presented in Table 1. No statistically significant difference was shown in shoulder sling wear time approximation among the three data logger locations (*p* > .05).

**Fig. 3:**
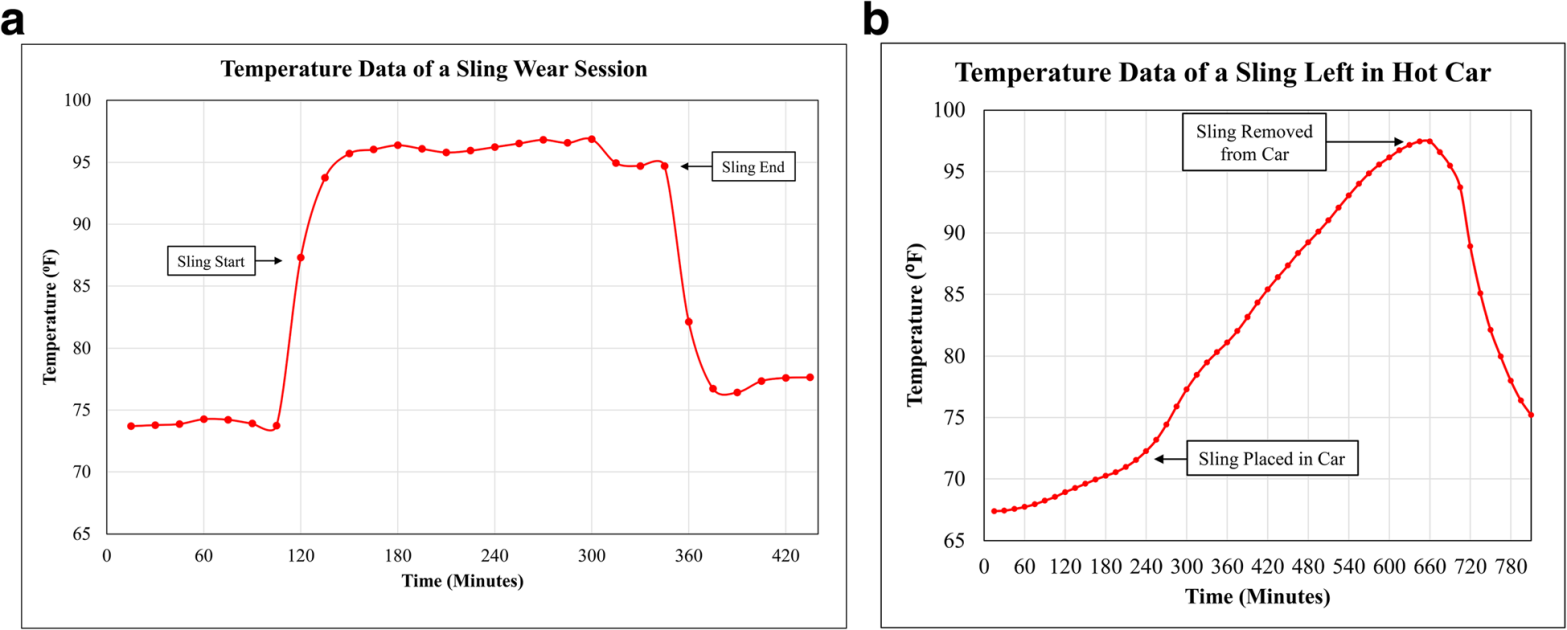
**a** Represents the temperature recording of actual sling wear. Note the steep change in temperature with the start of sling wear. **b** Shows the temperature curve of a sling left in a car on a hot day. Note the gradual temperature increase over more than 6 h

When the sling was left in the trunk of a car with outside temperatures ranging between 80 and 90 °F, it took 4.17 h for the data logger to equilibrate to a temperature of 85 °F before eventually peaking at 97 °F approximately 6.83 h later **(**Fig. 3a**)**. In comparison, when the sling was actually worn, the temperature reached 85 °F in <45 min **(**Fig. 3b**)**. The rate of temperature increase was 0.48°F per minute for actual sling wear, while it was 0.06°F per minute for the sling in a hot car. The rate of change was eight times faster for actual sling wear.

## Discussion

The results of the study support our hypothesis. The described protocol is an accurate method to measure patient compliance with shoulder sling wear. Although the placement of the data logger at the bolster location had the highest diagnostic accuracy of 99.5 %, no significant difference was shown among the three locations tested and all locations had an accuracy >99 %.

The World Health Organization has identified poor adherence as one of the major causes of failure to recover from long-term illnesses [13]. Cuff et al. [14] reported that poor adherence might be connected to worse functional outcomes in patients with rotator cuff repairs but did not assess for possible indicators of poor adherence beyond workers’ compensation status. Silverio et al. [10] evaluated the effects of social and demographic factors with patients’ self-reported adherence and functional outcomes by adherence measurement questionnaire in the rotator cuff repair population. However, that study might have been limited by recall bias and inaccurate responses. Morten et al. [10] showed that when back brace wear is accurately monitored, wear time as reported by the patient is unreliable.

An accurate measurement of recommended shoulder sling wear is an important research tool. Patients are instructed to wear a sling after rotator cuff surgery to avoid excessive strain on the repair, which can possibly decrease the incidence of re-tear. However, prolonged shoulder sling immobilization has been linked to shoulder stiffness [15]. Slings are often prescribed for consistent use for a six- to eight-week period, but no study to date has measured optimal sling wear time after shoulder surgery. Jenssen et al. [16] recently found that postoperative sling immobilization for three weeks was noninferior to six weeks of immobilization. Thus, our study validates a device that can be used to clarify the ambiguity surrounding appropriate postoperative sling-wearing time.

Previous studies in scoliosis and clubfoot research have shown patients do not accurately report their brace use [5, 11, 12]. Healthy volunteers with knowledge of the study aims were used to provide accurate self-reported logs (i.e., “actual wear time”) without fear of over- or under-reporting sling use. If surgical patients were used in this study, we would have asked them to wear their slings as much as possible to protect their repairs and prevent instability. Since no such concerns existed for the healthy volunteers, they could safely remove their slings to perform daily activities. This provided more variability in the temperature data to enable the creation of an algorithm to accurately detect the actual wear time with temperature sensors.

It is important to address the debate of the importance of sling wear postoperatively for rotator cuff tears. Tirefort et al. [17] recently showed no difference in outcomes when comparing sling immobilization versus no restrictions postoperatively for small-to-medium sized tears. Compliance with sling immobilization was self-reported, however, and may not reflect actual sling wear. Additionally, this study did not include larger tears where immobilization remains the recommendation in the immediate postoperative period.

The ambient temperature control test indicated the effects of a hot environment on the data loggers. The loggers differentiated the ambient heat of the hot environment from body temperature using our algorithm. Although the final temperatures reached by the sensor in the hot environment versus when the sling was worn were similar, the final temperature was met at a much slower rate (eight times slower) when the sensor was in a hot environment. This is likely caused by the differences in efficiency of conductive (i.e., actual sling wear) versus convective (i.e., sling in a hot car) heat transfer. Our algorithm is able to discern the difference in rates of temperature change to determine whether the shoulder sling is being worn. Implementation of the algorithm improves the accuracy of the loggers and decreases false positive readings.

The sample size of four patients in the present study was selected based on previous studies using temperature sensors to monitor brace use in patients with adolescent idiopathic scoliosis [5]. Each temperature sensor location in the present study recorded a total of 4405 temperature readings over a 1101.5-hour period. This results in a total of 13,215 temperature readings for all three temperature sensor locations. The minimum sample size for sensitivity studies ranges between 60 and 4860 tests performed [18]. Our study greatly exceeds this number and is powered to accurately screen for sling use. Additionally, a post-hoc power analysis for the correlation between the actual and measured sling wear showed the study achieved 84.4 % power despite the small sample size. This can be attributed to the near perfect correlation between these parameters due to the accuracy of the temperature sensors.

The accuracy of the data loggers might have been limited by the monitoring intervals used in our study. Although the sensor recorded temperatures at 15-minute intervals, the algorithm and data loggers can potentially underestimate the sling wear time by as much as 30 min per wearing session because of the intervals at which data is recorded. Repeatedly applying and removing the sling can account for a lower approximated wear time and thus affect the percent accuracy of the algorithm. Accuracy might be improved if smaller monitoring intervals for the data logger are used. The sensor on the palmar surface of the carpometacarpal joint was found to slightly overestimate wear time and had the highest overall estimated time. This is likely because the sensor in that location moves more freely and does not have as much intimate body contact. As a result, it is susceptible to greater fluctuations in temperatures. Finally, the accuracy of temperature sensors to monitor sling use can be potentially “cheated” by wearing the sling, then leaving it in a warm environment. If the environment allowed the temperature to rise above 100°F, however, the algorithm proposed in this study would detect that the sling was not actually being worn. The volunteers used in this study were aware of the study aims and were not penalized for wearing the sling less or incentivized to wear the sling more. Volunteers were only asked to be honest about their sling wear, as their sling wear logs were considered the actual sling wear time from which the algorithm was derived. The use of temperature sensors in future studies could be limited by “cheating” if the patients are aware of the temperature sensor, and the Hawthorne effect could alter their behavior if they know they are being monitored. Future studies will use the proposed algorithm to monitor sling use in actual patients.

## Conclusions

The described algorithm can effectively quantify shoulder sling wear time based on heat-generated sensor readings. False positives from ambient temperature are minimal. This method of accurately measuring sling wear could be used to study the relationship between postoperative sling use and functional outcomes after shoulder surgery.

## Supplementary Information

Additional file 1: **Appendix 1**. Algorithm outline.

Additional file 2: **Appendix 2**. Example data to illustrate the sling algorithm.

## Data Availability

Datasets used or analyzed in this study are available from the corresponding author upon request.

## Abbreviations

C: Celsius
F: Fahrenheit
