# Supplementary material for: An accurate method of measuring shoulder sling compliance: a validation study
Source: BMC Musculoskelet Disord. 2021 Jun 7;22:524. doi: 10.1186/s12891-021-04396-1 (PMC8186189; doi:10.1186/s12891-021-04396-1)
Supplement: Supplementary file 1 — Appendix 1. Algorithm outline. [file 12891_2021_4396_MOESM1_ESM.docx]

**Appendix 1. Algorithm Outline**

The following conditions must be met for a time point to be considered the start of a wear period:

1. The temperature must rise above 83°F, and either of the two options must be true:
   1. Option 1: The temperature increases ≥ 2°F between consecutive time points before crossing the 83°F threshold
      1. Set sling on time as time point immediately succeeding ≥ 2°F increase
   2. Option 2: Two consecutive increases ≥ 3°F between time points and the temperature increases above 83°F threshold within 30 minutes
      1. Set sling on time as time point immediately succeeding first ≥ 3°F increase
2. Temperature must remain ≥83°F for at least 30 minutes
3. Do not record sling wear if temperature exceeds 100°F

The following conditions must be met for a time point to be considered the end of a wear period:

1. The temperature must fall below 83°F
   1. And the temperature must decrease ≥ 3°F between time points within 30 minutes before crossing 83°F threshold
      1. Set sling off time as time point immediately before ≥ 3°F decrease
2. Must be during an established wear period
