# Supplementary material for: An accurate method of measuring shoulder sling compliance: a validation study
Source: BMC Musculoskelet Disord. 2021 Jun 7;22:524. doi: 10.1186/s12891-021-04396-1 (PMC8186189; doi:10.1186/s12891-021-04396-1)
Supplement: Supplementary file 2 — Appendix 2. Example data to illustrate the sling algorithm. [file 12891_2021_4396_MOESM2_ESM.docx]

**Appendix 2. Example Data to Illustrate the Sling Algorithm**

| **Example Data to Illustrate the Sling Algorithm** | |
| --- | --- |
| **Time** | **Temperature (⁰F)** |
| 00:00 | A |
| 00:15 | B |
| 00:30 | C |
| 00:45 | D |
| 01:00 | E |
| 01:15 | F |
| 01:30 | G |
| 01:45 | H |
| 02:00 | I |
| 02:15 | J |
| 02:30 | K |

*Application of the Sling Algorithm to Example Data*

For time 00:00 to be considered the start of a wear period, either option one or option two must be true:

1. Option 1:

- (B – A) ≥ 2⁰
- C ≥ 83⁰
- D ≥ 83⁰
- E ≥ 83⁰

1. Option 2:

- (B – A) ≥ 3⁰
- (C – B) ≥ 3⁰
- E ≥ 83⁰ or F ≥ 83⁰

For time 01:30 to be considered the end of a wear period, both condition one and condition two must be true:

1. Condition 1: (G – H) ≥ 3⁰
2. Condition 2: I ≤ 83⁰ or J ≤ 83⁰
